# Relationship between the Incidence of Dengue Virus Transmission in Traditional Market and Climatic Conditions in Kaohsiung City

**DOI:** 10.1155/2021/9916642

**Published:** 2021-08-09

**Authors:** Chung-Hao Huang, Chun-Yu Lin, Chun-Yuh Yang, Ta-Chien Chan, Po-Huang Chiang, Yen-Hsu Chen

**Affiliations:** ^1^Division of Infectious Disease, Department of Internal Medicine, Kaohsiung Medical University Hospital, Kaohsiung, Taiwan; ^2^School of Medicine, Graduate Institute of Medicine, Sepsis Research Center, Center for Tropical Medicine and Infectious Disease Research, Kaohsiung Medical University, Kaohsiung, Taiwan; ^3^Department of Public Health, College of Health Sciences, Kaohsiung Medical University, Kaohsiung, Taiwan; ^4^Research Center for Humanities and Social Sciences, Academia Sinica, Taipei, Taiwan; ^5^Institute of Public Health School of Medicine, National Yang Ming Chiao Tung University, Taipei, Taiwan; ^6^Institute of Population Health Sciences, National Health Research Institutes, Zhunan, Taiwan; ^7^Department of Biological Science and Technology, College of Biological Science and Technology, National Chiao-Tung University, Hsin-Chu, Taiwan

## Abstract

In 2014 and 2015, Southern Taiwan experienced two unprecedented outbreaks, with more than 10,000 laboratory-confirmed dengue cases in each outbreak. The present study was aimed to investigate the influence of meteorological and spatial factors on dengue outbreaks in Southern Taiwan and was conducted in Kaohsiung City, which is the most affected area in Taiwan. The distributed lag nonlinear model was used to investigate the role of climatic factors in the 2014 and 2015 dengue outbreaks. Spatial statistics in the Geographic Information System was applied to study the relationship between the dengue spreading pattern and locations of traditional markets (human motility) in the 2015 dengue outbreak. Meteorological analysis results suggested that the relative risk of dengue fever increased when the weekly average temperature was more than 15°C at lagged weeks 5 to 18. Elevated relative risk of dengue was observed when the weekly average rainfall was more than 150 mm at lagged weeks 12 to 20. The spatial analysis revealed that approximately 83% of dengue cases were located in the 1000 m buffer zone of traditional market, with statistical significance. These findings support the influence of climatic factors and human motility on dengue outbreaks. Furthermore, the study analysis may help authorities to identify hotspots and decide the timing for implementation of dengue control programs.

## 1. Introduction

Dengue fever is one of the most prevalent mosquito-borne infectious diseases worldwide and is caused by four distinct dengue virus (DENV) serotypes (DENV 1–4) [1]. Dengue virus belongs to the genus *Flavivirus* in the family Flaviviridae [2]. Approximately 2.5 billion people in more than 100 countries are at risk of contracting dengue virus, particularly in tropical and subtropical regions of the Southeast Asia and Western Pacific [3]. Approximately half of the world's human population is at a risk of dengue infection [4]. Dengue infection usually causes asymptomatic, flu-like symptoms; however, severe and fatal form, called dengue hemorrhagic fever, can cause bleeding, shock, and death [5]. There has been only one dengue vaccine (CYD-TDV) licensed until 2019, which was developed by Sanofi Pasteur [6, 7]. This vaccine which is only recommended for those people who have been previously infected with dengue virus, however, carries an increased risk of severe dengue in others [8, 9]. Therefore, avoiding mosquito bites remains the best way to prevent dengue virus infection and reduce its transmission.

It is predicted that the global mean temperature will rise by more than 1.5°C by the end of this century [10]. Rise in temperature will increase the frequency of dengue epidemics in Asian countries, along with spread to new geographic regions [11]. Environmental risk factors may contribute to the geographic expansion of dengue [12, 13]. Multiple environmental risk factors are known to influence dengue virus transmission, including ineffective vector control operations, climatic conditions, temperature, precipitation, humidity, and population movements [14]. Climatic conditions and temperature affect the life cycle of *Aedes* mosquitoes, including larval development and adult survival, length of the gonotrophic cycles, and distribution [15, 16]. The distribution of dengue vectors corresponds to the virus epidemic. The predominance of *Aedes aegypti* mosquitoes and dengue epidemics or outbreaks in Southern Taiwan is highly correlated [17]. A majority of dengue outbreaks reported in the recent decades were reported in locations south of the Tropic of Cancer, which geographically divides the island of Taiwan into two climatic regions, tropical and subtropical [18]. Climate change is one of the main reasons for this phenomenon; only an environment with a tropical climate is suitable for mosquito transmission of dengue virus. The whole Taiwan island reported outbreaks of dengue fever in the years of 1915, 1931, and 1942. Dengue outbreaks of varying degrees occurred in Southern Taiwan every year after 1989 [17, 19]. A large and historical dengue outbreak occurred in Kaohsiung City in 2002, and the number of laboratory-confirmed dengue cases reached 5336, including 241 cases of dengue hemorrhagic fever, which caused 19 deaths [20, 21]. In 2014 and 2015, unprecedented dengue outbreaks occurred in Southern Taiwan. In 2014, a total of 15,492 laboratory-confirmed dengue cases were reported in Kaohsiung City, accounting for 96% of 15,732 cases reported by the Centers for Disease Control, Taiwan (Taiwan CDC) [22]. In 2015, consecutive dengue outbreak originated in Tainan City and then spread to Kaohsiung City, and a total of 43,784 dengue cases, mostly distributed in Tainan (52%) and Kaohsiung (45%), were reported by the Taiwan CDC [22].

*A. aegypti* and *A. albopictus* are two main dengue vectors present in Kaohsiung City. The favorable breeding sites for *Aedes* mosquitos are artificial containers, such as water tanks, tires, flower pots, flower vases, water trays, storage tanks, and discarded containers. On the contrary, *A. albopictus* breeds preferentially in natural containers, such as tree holes and bamboo tubes. The most effective strategy for dengue control and management is cleaning of breeding places. Traditional markets can facilitate transmission of some infectious diseases and increase the risk of outbreak; for example, influenza A outbreaks in farmer/agricultural markets have been reported [23]. The agricultural markets may play an important role in the transmission of zoonotic diseases [24]. In addition to influenza, Pabilonia et al. reported that *Salmonella* spp. is abundant in the environment [25]. In wet markets, plenty of water is used for cleaning, and water logging is common, which may facilitate mosquito breeding.

To understand the behavior of dengue virus transmission, investigation of climate space-time characteristics and other risk factors is important. Several previous studies have demonstrated that space-time dynamics is important for *Aedes* mosquito development and dengue virus transmission [20, 26]. Relationships between meteorological factors and dengue fever prevalence, particularly the delayed temporal lags with respect to weather variables, are critical for the early warning system of dengue virus epidemics [24, 27]. Few studies have investigated the epidemiology of dengue virus and the lag effect of climate variables on dengue transmission in Kaohsiung City [24, 27]. The distribution lag nonlinear model (DLNM) is a flexible model to examine the delayed lag effect of different climatic variables on dengue incidence [28, 29]. The DLNM is based on a cross-basis function that examines a two-dimensional relationship along the dimensions of climate change, temperature, rainfall, and time lag in weeks [28, 30, 31]. Taiwan has experienced the worst outbreak of mosquito-borne dengue fever in decades and a record hot summer in 2014-2015; therefore, the frontline public health workers are in urgent need of a strategy to control the transmission of the disease. In this study, we investigated the influence of ecology and human dispersal on dengue outbreaks in Kaohsiung City. DLNM was used to investigate the association of delayed effects of the selected meteorological conditions with weekly recorded dengue cases between 2014 and 2015 in Kaohsiung City. Moreover, the Geographic Information System (GIS) tool was used to provide necessary information on hotspot areas for the outbreak and populations at the highest risk of contracting the disease.

## 2. Materials and Methods

### 2.1. Study Area and Data

**Figure 1:**
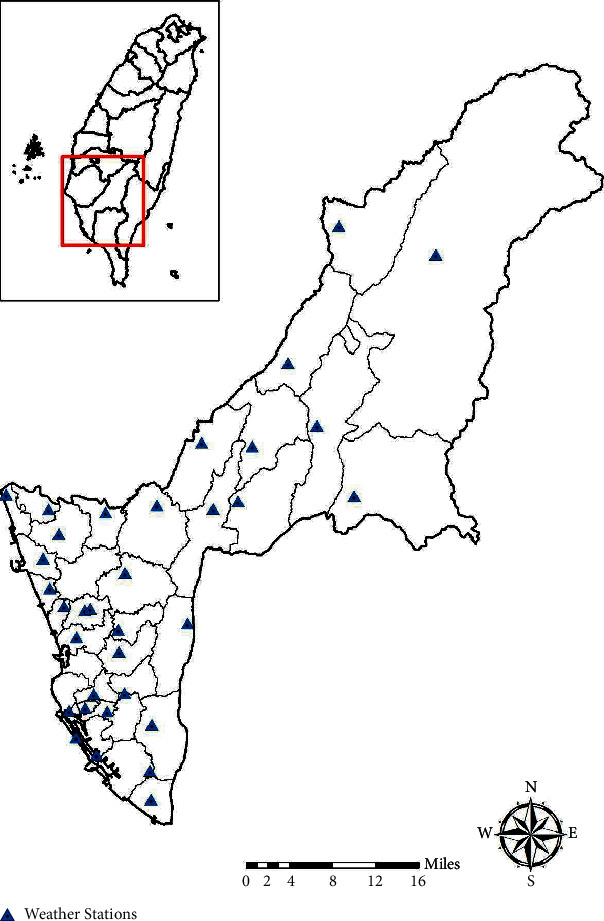
Map of Kaohsiung City, Taiwan (22^o^38′*N*, 120^o^16′*E*). The city is divided into 38 districts. The locations of automatic observation stations and weather stations of the Kaohsiung City are shown (▲).

Dengue case data were collected from the government's open data portal. The dataset included the number of daily confirmed cases from 2014 to 2015, illness onset date, living city/county, township and village, basic statistical area, central point *x*-axis and *y*-axis of the basic statistical area, and latitude and longitude coordinates. There were 34,817 confirmed dengue cases in the dataset, and all were enrolled in the ecological analysis. In recent years, most dengue outbreaks occurred in Kaohsiung City, Southern Taiwan, and its 38 districts were considered as the areas of interest in this study, and 17,497 basic statistical areas were used to make data match the original case distribution. All epidemiological data were obtained from the Taiwan CDC's surveillance database. Weekly meteorological data of every district were obtained from the Central Weather Bureau and the Water Resource Agency's weather monitoring stations. Meteorological data, including daily average temperature and precipitation, were collected from 38 weather stations in Kaohsiung City (Figure 1). In the study of dispersal, 34,817 dengue cases were enrolled and analyzed. Using the Kriging method, the values of township were estimated in weeks. The 1997 Taiwan Datum (TWD 97) coordinate system with a county/township layer and point of information of traditional markets in Kaohsiung in 2013 was used. The electronic map used a scale of 1 : 5000.

### 2.2. Distributed Lag Nonlinear Model (DLNM)

The mosquito life cycle is affected by the weather conditions [32, 33]. To understand the effect of weather on the dengue outbreak, the lag effect of weather needs to be considered. Thus, we developed a DLNM model to simultaneously assess the nonlinear temporal lagged effects of meteorological factors and geographical heterogeneity on the spatiotemporal distribution of dengue fever incidences. We assume that Ydt represents the number of weekly dengue fever cases at calendar time (week) *t* ∈ (0, 2,…, 52) in district *d* ∈ (1, 2,…, 38) and follows a Poisson distribution by *Y*_*dt*_ | *μ*_*dt*_ ∼ POI (*μ*_*dt*_), where *μ*_*dt*_ is the expected value of *Y*_*dt*_. Hence, a DLNM was established based on a geoadditive structure with a quasi-Poisson family as follows:

(1) $$\begin{array}{c} \log\left(\mu_{dt}\right)=\alpha +f\left(\text{Time}\right)+f\left(TP,\text{lag}=20\right)+f\left(RF,\text{lag}=20\right)+f_{\text{spac}}\left(d\right)+\text{offset}, \end{array}$$

where *α* is the intercept. The time smoother *f* (Time) with respect to the week is a cubic spline for controlling temporal autocorrelations. The effect of meteorological variation was investigated via two cross-basis functions *f* (*TP*, lag) and *f* (*RF*, lag), to describe the association between the space of temperature (TP) and rainfall (RF) with an average temporal lag of 20 weeks. The choice of 20 weeks was based on the potential delayed periods of meteorological effects in previous studies [34]. Each rainfall variable was log-transformed before fitting into the model. The spatial function f_spac_ (*d*) adopts Markov random fields to adjust spatial autocorrelations and describe geographic heterogeneity. The geographical heterogeneity f_spac_ (*d*) denotes the spatial variations of dengue fever cases, which cannot be explained by meteorological variables. The Markov random field of f_spac_ (*d*) is achieved by a conditional autoregressive prior with a normal distribution with a mean of ∑_*d*′∈Ω_*φ*_*d*′_/*N*_*d*_ and a variance of *σ*_*d*_^2^/*N*_*d*_. The neighborhood set as Ω contains all the adjacent districts, which have overlapped boundaries designated as “*d*.” The spatial effect of an adjacent district “*d*” is denoted by *φ*_*d*′_, and *N*_*d*_ is the number of adjacent districts near district “*d*.” In other words, the spatial function is a function of districts, which accounts for the logarithm of relative risks across the districts within the study area and considers the spatial autocorrelation of incidence under the Markov random-fields framework. The estimation of spatial function under the generalized additive structure was based on the Markov chain Monte Carlo algorithm [35]. The offset is the logarithm of the district-level population, averaged from the annual census data from 2014 to 2015. The averaged data were used because of the limited change in the population sizes of every district over the study area during the study period. Data analysis was conducted using the *R* version 2.14.1 (R Development Core Team, 2001) and SAS v9.3 (SAS Institute Inc., Cary, NC, USA). Statistical significance was determined at 95% confidence interval (CI).

### 2.3. Geographic Information System

To elucidate the relationship between the spread of dengue outbreaks and human motility (traditional market), the empirical Bayesian Kriging (EBK) was used to estimate the distribution of dengue cases spreading (transform data from point to polygon). The effect of distance was considered, and the Inverse Distance Weighted (IDW) method was used to estimate the distribution of dengue cases. Then, the distribution map was overlayed with a map of traditional markets in Kaohsiung City. To investigate the effect of the traditional market on dengue case distribution, the buffer analysis was applied, considering traditional market as a center to establish a buffer zone of 1,000 m (10-min walking time). Regression analysis was used to investigate the relationship between the number of traditional markets and dengue cases. All analyses were performed using the ArcGIS Desktop 10.1.

## 3. Results

**Figure 2:**
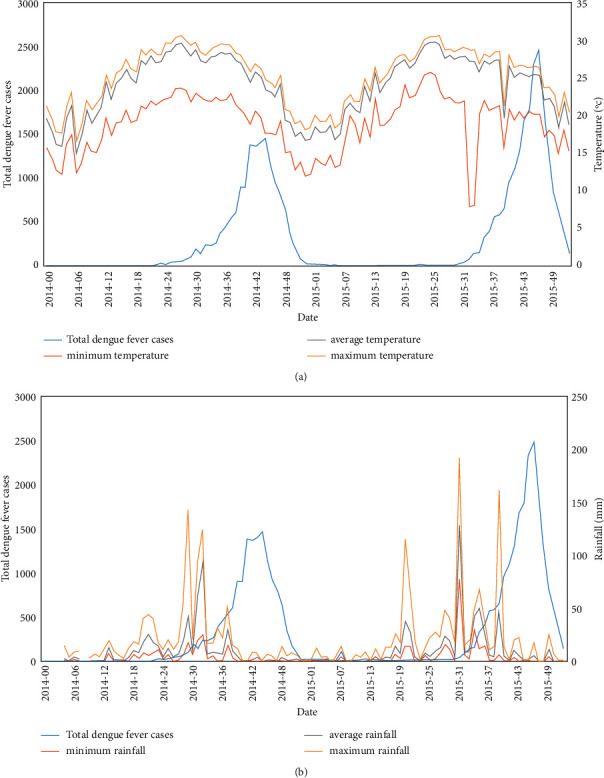
Time-series weekly average temperature (a) and rainfall measures (b) and total dengue fever cases from 2014 to 2015 in Kaohsiung City.

Meteorological factors have been shown to be important for the space-time dynamics of dengue fever transmission. To develop a disease warning system, it is essential to understand the empirical relationship between meteorological factors and dengue fever. During the study period (January 2014–December 2015), the Taiwan CDC reported 34,817 laboratory-confirmed dengue cases in 38 districts of Kaohsiung City. The locations of the automatic observation stations and weather stations are shown in Figure 1. The annual onset of epidemics generally coincided with the peak in monsoon rainfall and temperature level (Figure 2).

### 3.1. Relative Risk of Dengue Fever with Temperature and Rainfall

**Figure 3:**
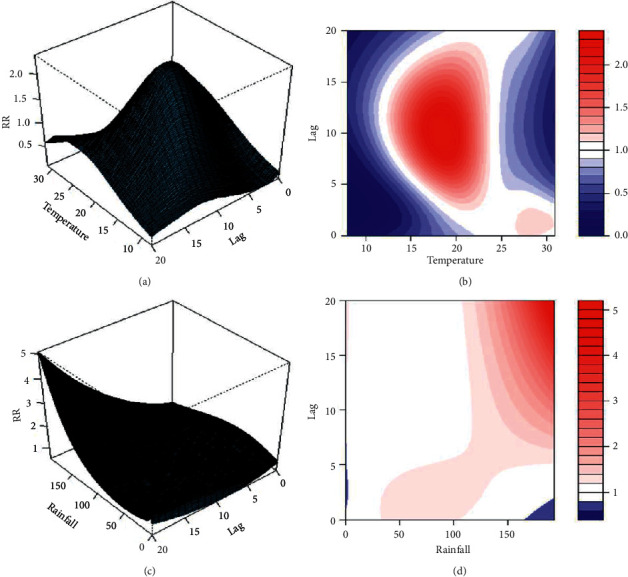
The three-dimensional graphs and corresponding contour plots showing the relative risk of dengue fever incidence at lagged weeks along with temperature (a), (b) and rainfall (c), (d).

The relative risk (RR) of dengue fever with respect to average temperature and total precipitation with different lags is represented by three-dimensional graphs and contour plots, as shown in Figure 3. The average temperature of 24.25°C and rainfall of 9.49 mm were defined as references for RR. The RR of dengue fever peaked after a lag in temperature (Figure 3(a)). As shown in the contour plot, the data reveal a considerable change in the RR at different temperatures and lags (Figure 3(b)). The RR of dengue fever was above 1.5, when the weekly average temperature was between 14°C and 25°C at lagged weeks 5 to 15. The RR reached a peak of 2.37, when the average temperature was 18.5°C.

Higher RR of dengue was observed at greater weekly average precipitation and more lagged weeks (Figure 3(c)). After lagged week 4, the RR of dengue incidence increased with increase in rainfall, as shown in the contour plot of the three-dimensional graph (Figure 3(d)). The RR reached 3 after lagged week 12 and reached the maximum to 5.08 at lagged week 20 (Figure 3(d)).

### 3.2. Alterations in the Relative Risk of Dengue Virus Infection in Different Lagged Weeks of Average Temperature

**Figure 4:**
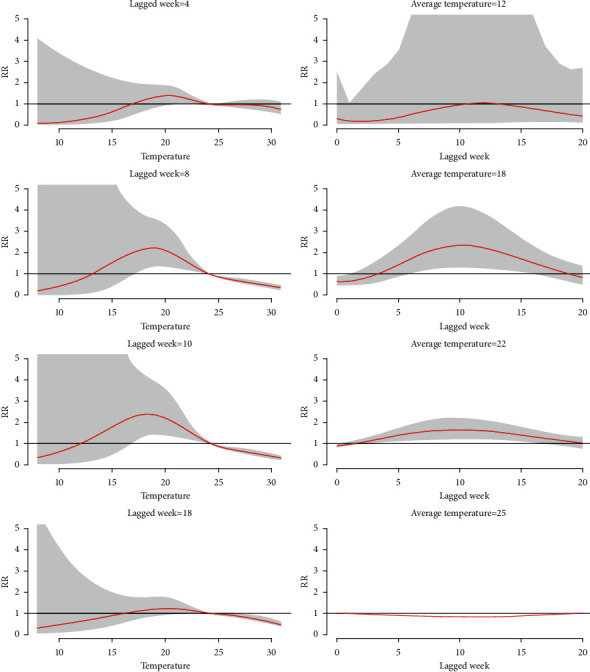
The estimation of relative risk of dengue fever associated with minimum temperature at selected lagged weeks (a) and lagged weeks with respect to selected minimum temperature (b). The shaded region indicates 95% confidence interval.

Figure 4(a) reveals that, at specific lagged weeks [4, 8, 10, 18], the RR of dengue incidence changes corresponding to weekly average temperature. Figure 4(b) displays that, at specific average weekly temperatures (12°C, 18°C, 22°C, and 25°C), the RR response alters in different lagged weeks. The RR of dengue fever was approximately 1 at average weekly temperatures of 12°C and 25°C at different lagged weeks, as shown in Figure 4(b). When the average weekly temperature was above 12°C, the RR of dengue fever increased, particularly at lagged weeks 5 to 15 (Figure 3(b) and 3(d)). However, the RR decreased at average weekly temperature of more than 18°C in different lagged weeks, as shown in Figure 4(a). Figure 4(a) shows that the RR of dengue fever increased after lagged week 4 and started to drop after lagged week 10.

### 3.3. Alterations in the Relative Risk of Dengue Virus Infection in Different Lagged Weeks of Rainfall

**Figure 5:**
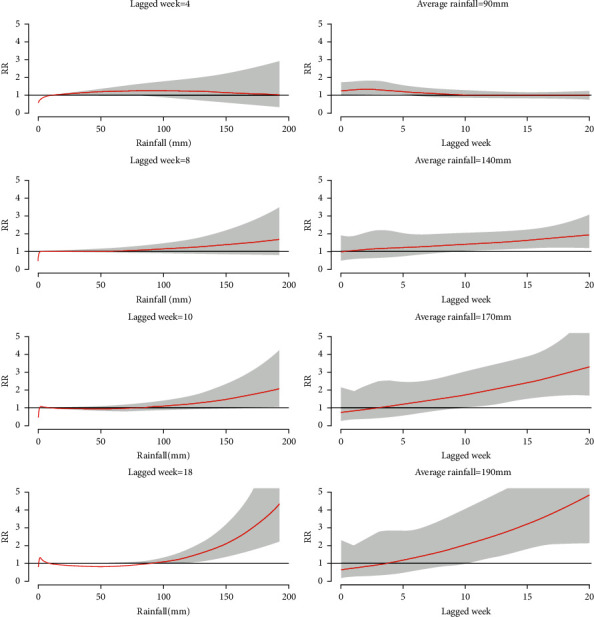
Relative risk of dengue fever associated with rainfall at selected lagged weeks (a) and lagged weeks with respect to selected rainfall (b). The shaded region indicates 95% confidence interval.

Figure 5 displays the changes in the RR of dengue fever corresponding to different average weekly rainfall and lagged weeks at specific lagged weeks [4, 8, 10, 18] and rainfall (90, 140, 170, and 190 mm), respectively. When the average rainfall was less than 90 mm, the RRs of dengue fever in most lagged weeks were approximately 1. However, the RR of dengue infection increased gradually when the average weekly rainfall was more than 140 mm (Figure 5(b)).

### 3.4. Traditional Market Is a Relative Risk of Dengue Virus Infection

**Figure 6:**
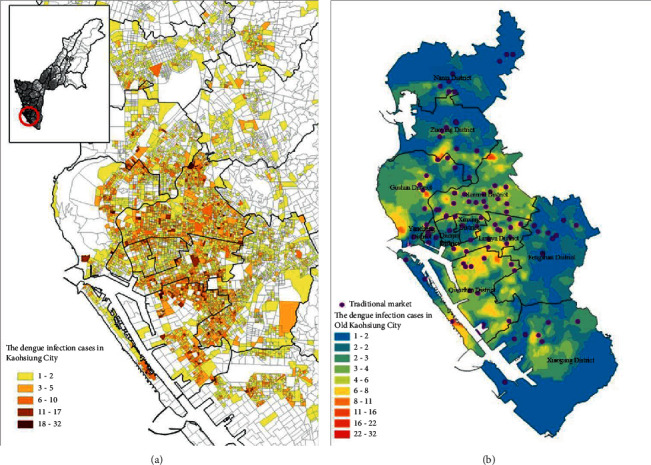
The dengue infection cases in Kaohsiung City in 2014–2015 (a). The locations of the traditional market of Kaohsiung City (b).

The number of dengue cases was estimated by the EBK model, and the distribution of the cumulative number of cases is shown in Figure 6(a); deeper color represents greater number of cases. The cases were mainly concentrated in the old Kaohsiung City (Figure 6(a)). The distribution of hotspots of dengue cases in old Kaohsiung City was corrected with the locations of traditional markets after map overlay (Figure 6(b)). The regression analysis also revealed a significant correlation between the number of dengue cases and traditional markets (Table 1). In order to understand the epidemic spreading pattern, a buffer zone of 1000 m (waking time of approximately 10 minutes for adults) was established considering traditional markets as centers, and the percentage of cases in the buffer zone (cumulative number of cases in the buffer zone/cumulative number of cases in the administrative district, where the corresponding traditional market was located) was calculated. The buffer analysis in the GIS revealed that the cumulative number of dengue cases in the buffer zone was 15,206, whereas in the corresponding administrative district, it was 18,225, showing that approximately 83% of the cases were located in the buffer zone of 1000 m around traditional markets.

## 4. Discussion

Many studies have shown that climatic conditions such as temperature and rainfall are strongly associated with dengue transmission. The global burden of dengue fever has increased rapidly in recent years. Many countries in tropical and subtropical areas have experienced annual outbreaks. Kaohsiung City is the center of major dengue virus epidemics in Taiwan. The climatic factors (temperature and rainfall) were investigated for being the potential risk factors of dengue outbreaks using the DLNM. The results showed that weekly average temperatures of more than 15°C at lagged weeks 5 to 18 were the most significant variables associated with an increase in the RR of dengue fever. The nonlinear relationship between average rainfall and dengue epidemics was related to the precipitation effects on the lifecycle of female mosquitoes. Rainfall provides an environment for breeding habitats suitable for the proliferation of *Aedes* spp. mosquitos and favors an increase in the number of mosquito eggs hatching. The elevated RR of dengue was observed when the weekly average rainfall was more than 150 mm at lagged weeks 12 to 20. The risk of dengue infection increased during the rainy season when *the Aedes* mosquito infestation reached its peak. Artificial containers, if not frequently emptied, act as breeding sites for vectors. Our findings demonstrated that the mosquito population was mainly driven by temperature and rainfall, affecting the dengue fever epidemics in Taiwan.

Additionally, the relationship between human motility (location of traditional market) and dengue spreading pattern was studied using the geostatistical analysis in the GIS. It has been observed that the level of urbanization was a leading factor in elevating the risk of dengue fever in Taiwan. The location with higher population density was associated with higher dengue fever incidence. This finding suggests that *A. aegypti* is the primary vector responsible for dengue fever epidemics in Taiwan since urban areas are generally preferred habitats for *A. aegypti*.

## 5. Conclusions

The findings in this study support previous studies showing a considerable correlation between precipitation and temperature and the occurrence of dengue virus epidemics in Southern Taiwan. This study shows that the annual onset of dengue virus epidemics coincides with the time of peak rainfall and temperature. Moreover, dengue virus outbreaks coincide with the seasonal increase in *Aedes* mosquito population in the traditional market environment. These findings support the influence of climatic factors and human motility on dengue outbreaks. Furthermore, the study analysis may help authorities to identify hotspots and decide the timing for implementation of dengue control programs.

## Figures and Tables

**Table 1:** Regression analysis of dengue infection cases and traditional markets.

	Coefficient	*p* value
Kaohsiung City		
Intercept	−53.02	0.443
Number of traditional markets	192.15	<0.001
Old Kaohsiung City		
Intercept	−748.3	0.0357^*∗*^
Number of traditional markets	248.1	<0.001

## Data Availability

The data used to support the findings of this study are included within the article.

## References

[1] Guzman M. G., Harris E. (2015). Dengue. *The Lancet*.

[2] Chambers T. J., Hahn C. S., Galler R., Rice C. M. (1990). Flavivirus genome organization, expression, and replication. *Annual Review of Microbiology*.

[3] Gubler D. J., Clark G. G. (1995). Dengue/dengue hemorrhagic fever: the emergence of a global health problem. *Emerging Infectious Diseases*.

[4] Rigau-Pérez J. G., Clark G. G., Gubler D. J., Reiter P., Sanders E. J., Vance Vorndam A. (1998). Dengue and dengue haemorrhagic fever. *The Lancet*.

[5] Bhatt S., Gething P. W., Brady O. J. (2013). The global distribution and burden of dengue. *Nature*.

[6] Coronel-MartÍnez D. L., Park J., López-Medina E. (2021). Immunogenicity and safety of simplified vaccination schedules for the CYD-TDV dengue vaccine in healthy individuals aged 9-50 years (CYD65): a randomised, controlled, phase 2, non-inferiority study. *The Lancet Infectious Diseases*.

[7] Deng S. Q., Yang X., Wei Y., Chen J. T., Wang X. J., Peng H. J. (2020). A review on dengue vaccine development. *Vaccines*.

[8] Capeding M. R., Tran N. H., Hadinegoro S. R. S. (2014). Clinical efficacy and safety of a novel tetravalent dengue vaccine in healthy children in Asia: a phase 3, randomised, observer-masked, placebo-controlled trial. *The Lancet*.

[9] Hadinegoro S. R., Arredondo-García J. L., Capeding M. R. (2015). Efficacy and long-term safety of a dengue vaccine in regions of endemic disease. *New England Journal of Medicine*.

[10] Pfleiderer P., Schleussner C., Mengel M., Rogelj J. (2018). Global mean temperature indicators linked to warming levels avoiding climate risks. *Environmental Research*.

[11] Yang H. M., Macoris M. L. G., Galvani K. C., Andrighetti M. T. M., Wanderley D. M. V. (2009). Assessing the effects of temperature on dengue transmission. *Epidemiology and Infection*.

[12] Reiter P. (2001). Climate change and mosquito-borne disease. *Environmental Health Perspectives*.

[13] Hii Y. L., Rocklöv J., Ng N., Tang C. S., Pang F. Y., Sauerborn R. (2009). Climate variability and increase in intensity and magnitude of dengue incidence in Singapore. *Global Health Action*.

[14] Welty L. J., Zeger S. L. (2005). Are the acute effects of particulate matter on mortality in the National Morbidity, Mortality, and Air Pollution Study the result of inadequate control for weather and season? A sensitivity analysis using flexible distributed lag models. *American Journal of Epidemiology*.

[15] Lifson A. (1996). Mosquitoes, models, and dengue. *The Lancet*.

[16] Yang H. M., Macoris M. L. G., Galvani K. C., Andrighetti M. T. M., Wanderley D. M. V. (2009). Assessing the effects of temperature on the population of *Aedes aegypti*, the vector of dengue. *Epidemiology and Infection*.

[17] Chang S.-F., Huang J.-H., Shu P.-Y. (2012). Characteristics of dengue epidemics in Taiwan. *Journal of the Formosan Medical Association*.

[18] Chen M. J., Lin C. Y., Wu Y. T., Wu P. C., Lung S. C., Su H. J. (2012). Effects of extreme precipitation to the distribution of infectious diseases in Taiwan. *PLoS One*.

[19] Wang S.-F., Chang K., Lu R.-W. (2015). Large Dengue virus type 1 outbreak in Taiwan. *Emerging Microbes & Infections*.

[20] Wen T.-H., Lin N. H., Chao D.-Y. (2010). Spatial-temporal patterns of dengue in areas at risk of dengue hemorrhagic fever in Kaohsiung, Taiwan, 2002. *International Journal of Infectious Diseases*.

[21] Lin C.-C., Lin Y.-S., Huang Y.-H. (2010). Characteristic of dengue disease in Taiwan: 2002-2007. *The American Journal of Tropical Medicine and Hygiene*.

[22] Wang S.-F., Chang K., Loh E.-W. (2016). Consecutive large dengue outbreaks in Taiwan in 2014-2015. *Emerging Microbes & Infections*.

[23] Morgan A. (2006). Avian influenza: an agricultural perspective. *The Journal of Infectious Diseases*.

[24] Fraga J., Botelho A., Sá A., Costa M., Quaresma M. (2011). The lag structure and the general effect of ozone exposure on pediatric respiratory morbidity. *International Journal of Environmental Research and Public Health*.

[25] Pabilonia K. L., Cadmus K. J., Lingus T. M. (2014). EnvironmentalSalmonellain agricultural fair poultry exhibits in Colorado. *Zoonoses and Public Health*.

[26] Tsuda Y., Suwonkerd W., Chawprom S., Prajakwong S., Takagi M. (2006). Different spatial distribution of *Aedes aegypti* and *Aedes albopictus* along an urban-rural gradient and the relating environmental factors examined in three villages in northern Thailand. *Journal of the American Mosquito Control Association*.

[27] Chen S.-C., Liao C.-M., Chio C.-P., Chou H.-H., You S.-H., Cheng Y.-H. (2010). Lagged temperature effect with mosquito transmission potential explains dengue variability in southern Taiwan: insights from a statistical analysis. *The Science of the Total Environment*.

[28] Gasparrini A., Armstrong B., Kenward M. G. (2010). Distributed lag non-linear models. *Statistics in Medicine*.

[29] Yang J., Ou C. Q., Ding Y., Zhou Y. X., Chen P. Y. (2012). Daily temperature and mortality: a study of distributed lag non-linear effect and effect modification in Guangzhou. *Environmental Health: A Global Access Science Source*.

[30] Shang C. S., Fang C. T., Liu C. M., Wen T. H., Tsai K. H., King C. C. (2010). The role of imported cases and favorable meteorological conditions in the onset of dengue epidemics. *PLoS Neglected Tropical Diseases*.

[31] Chen S.-C., Hsieh M.-H. (2012). Modeling the transmission dynamics of dengue fever: implications of temperature effects. *The Science of the Total Environment*.

[32] Descloux E., Mangeas M., Menkes C. E. (2012). Climate-based models for understanding and forecasting dengue epidemics. *PLoS Neglected Tropical Diseases*.

[33] Karim M. N., Munshi S. U., Anwar N., Alam M. S. (2012). Climatic factors influencing dengue cases in Dhaka city: a model for dengue prediction. *Indian Journal of Medical Research*.

[34] Chien L.-C., Yu H.-L. (2014). Impact of meteorological factors on the spatiotemporal patterns of dengue fever incidence. *Environment International*.

[35] Li R., Englehardt J. D., Li X. (2012). A gradient Markov chain Monte Carlo algorithm for computing multivariate maximum likelihood estimates and posterior distributions: mixture dose-response assessment. *Risk Analysis*.

